# François Rabelais and his dystonic giants

**DOI:** 10.1055/s-0044-1786764

**Published:** 2024-05-13

**Authors:** Léo Coutinho, Carlos Henrique Ferreira Camargo, Hélio Afonso Ghizoni Teive

**Affiliations:** 1 Hospital de Clínicas, Departamento de Medicina Interna, Programa de Pós-Graduação em Medicina Interna, Curitiba PR, Brazil.; 2 Hospital de Clínicas, Departamento de Medicina Interna, Serviço de Neurologia, Unidade de Distúrbios de Movimento, Curitiba PR, Brazil.

**Keywords:** History of Medicine, Neurology, Movement Disorders, Dystonia, Torticollis, História da Medicina, Neurologia, Transtornos dos Movimentos, Distonia, Torcicolo

## Abstract

*Spasmodic torticollis* was an early designation used for cervical dystonia. The origin of this name is attributed to French physician and writer François Rabelais in the mid-sixteenth century. This early description of torticollis in the book *Pantagruel* was an inspiration for the understanding of cervical dystonia. The art expressed in Rabelais' literature ‒ which was immortalized by the drawings of Gustave Doré ‒ influenced poetry, art, and photography, and led to the adoption of the term *torticollis* in the neurological sciences.

## Resumo

Uma designação inicial usada para distonia cervical era *torcicolo espasmódico* . A origem desse termo é atribuída ao médico e escritor francês François Rabelais em meados do século XVI. Essa descrição inicial do torcicolo no livro *Pantagruel* foi uma inspiração para a compreensão da distonia cervical. A arte exibida na literatura de Rabelais ‒ imortalizada pelos desenhos de Gustave Doré ‒ influenciou a poesia, a arte e a fotografia, e levou à adoção do termo *torcicolo* nas ciências neurológicas.

## INTRODUCTION

Dystonia is a movement disorder characterized by sustained or intermittent muscle contractions, causing abnormal, often repetitive, movements and/or postures. Dystonic movements are typically patterned, twisting, and may be tremulous. 1 The term *dystonia* was coined by Hermann Oppenheim in 1911. 2 In Latin, *tonus* means “stretching”, “quality of sound”, “tone”, or “accent”. This word was in turn derived from the Greek word *tonos* , which is also translated as “stretching”, or “tension”. 3 4 When dystonia affects the neck muscles, we refer to it as *cervical dystonia* . 4

The first description that resembles cervical dystonia dates to Classical Antiquity. Hippocrates reported a case of *traxhlos sklhros* (“stiff and painful neck”), an illness characterized by contraction of the jaws and cervical musculature, with a fatal course, possibly tetanus or meningitis. This phenomenology was later referred to by Celsus and Pliny the Elder by the term *rigor cervicis* . 5

Another early designation used for cervical dystonia was *spasmodic torticollis* . The origin of this term is attributed to French physician and writer François Rabelais (1483?–1553) in the mid-16th century. 5 6

**Figure 1:**
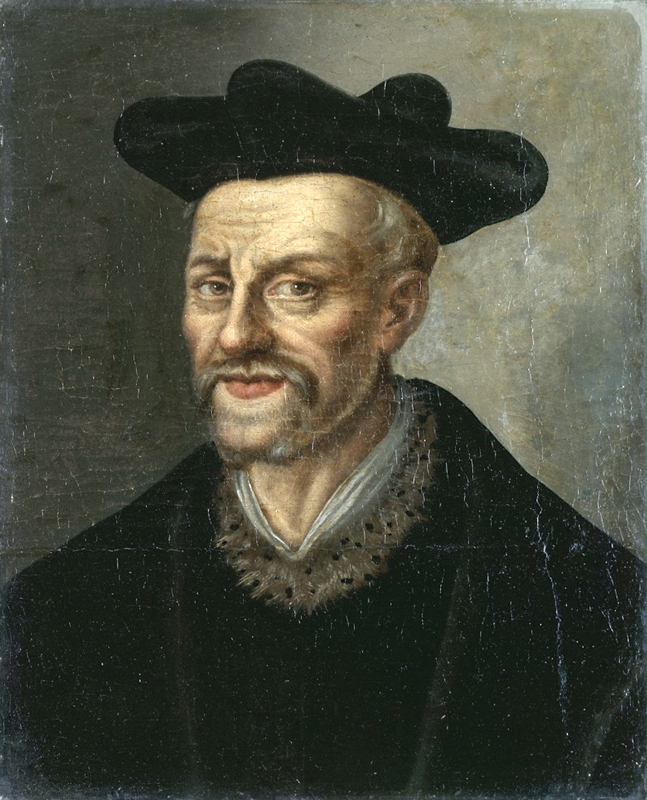
François Rabelais (1483?–1553). Licensed under a public domain mark.

The present paper reviews the contribution of Rabelais ( Figure 1 ) to the origins of the term *torticollis* , one of the first phenomenological terminologies widely adopted to designate cervical dystonia.

## FRANÇOIS RABELAIS: A REBEL GENIUS

Data on the life of Rabelais is very scarce. Born possibly in the year 1483 in Chinon, France, Rabelais seems to have started a law education but then initiated a religious career, spending ∼ 20 years as a Franciscan friar and a Benedictine monk. During his time within the Franciscan order, he corresponded with several members of the humanistic movement, such as William Budé. His transition to the Benedictine order occurred after he petitioned it to the Pope, given that the Benedictine order was more devoted to culture. There he resumed his law studies but also had his first contact with the field of medicine. 6 7

He received initial medical training at the Benedictine hospital of Saint-Denis, and eventually abandoned the clerical life to study medicine at the University of Paris, receiving his medical degree in 1530 in Montpellier and his doctorate 7 years later. He was appointed physician to the prestigious Hôtel-Dieu Hospital, in Lyon. At this time, he entered the inner circle of the eminent Du Bellay clan, serving the brothers Guillaume and Jean Du Bellay as their personal physician, secretary, and possibly diplomatic agent. 6 7

His writing occurred in consonance with his medical training, with the publication of *Pantagruel* in 1532, followed by *Gargantua* in 1534. His work presented a high degree of criticism toward the Church, often including vulgar predicates and insults. He published the first two books under the *nom de plume* Alcofrybas Nasier, an anagram of his own name, but assumed authorship of the *Third Book* , published in 1546, and the *Fourth Book* , published in 1552. The *Fifth Book* was published posthumously in 1564, but his authorship is a matter of debate, and it may be an unfinished draft by Rabelais polished and completed by another undisclosed author. 6 7

Due to the satirical nature of his work, Rabelais was persecuted by the Church. His works were censored by the Sorbonne and marked in the *Index Librorum Prohibitorum* (“Index of Forbidden Books”) as heretic. After the publication of the *Third Book* , Rabelais was forced to take refuge in Metz and later in Rome, where it is very likely only avoided imprisonment and condemnation due to the prestige of the Du Bellay brothers and other of his eminent patrons. 6 7

## THE ORIGIN OF TORTICOLITIS

In *Pantagruel* , Rabelais describes the miraculous healing of Epistemon, Pantagruel's tutor, who was decapitated. His head was sewed back on by Panurge, and the healer applied a cataplasm “so that he might not be wry-necked,” or, in the original French: “ *afin qu'il ne fust torty colly* .” The previously unheard word *torticollis* was therefore inspired by Rabelais, a writer known for his neologisms. 4 5 8

The word *torticollis* was then used by the French poet Paul Scarron, in the seventeenth century, and subsequently entered the medical vocabulary to describe patients presenting cervical dystonia with maintained posture to the sides of the neck 5 8 9

*Mon pauvre corps est raccourci* [“My poor body is shortened”]

*Et j'ai la tête sur I'oreille* [“And I have my head on my ear”]

*Mais cela me sied à merveille* [“But it suits me marvelously”]

*Et parmi les****torticollis*** [“And among the stiff-necked”]

*Je passe pour des plus jolis* [“I pass for one of the prettiest”]

**Figure 2:**
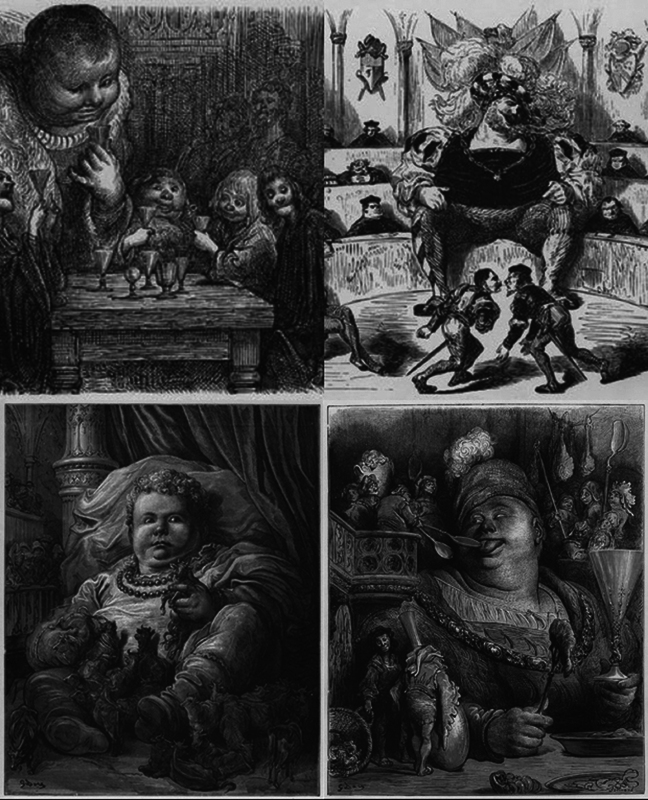
*Gargantua and Pantagruel* , by Gustave Doré. Licensed under a public domain mark.

In 1854, a version of Rabelais' work was published with drawings by the famous illustrator Gustave Doré. 10 It is interesting to note that, despite no other mention of *torty colly* , most of Doré's pictures represent both Gargantua and Pantagruel with their heads tilted to one side, suggesting some form of cervical dystonia in the infamous giants, possibly with a genetic pathogenesis ( Figure 2 ).

By the time of the publication of this edition illustrated by Doré, cervical dystonia had been extensively studied in the field of neurology for several years. The term *torticollis* was used in the works by Felix Plater (1536–1614), Nicolaas Tulp (1593–1674), and the seminal thesis by Georg Friedrich von Jäger (1714–1787), entitled *Caput obstipum affectum rariorem in libris et praxis* (“A Rarely Encountered Condition of Stiffened Neck in Books and Practice”). Descriptions of torticollis were also presented by Charles Bell, Moritz Romberg, Wilheim Erb, and Guillaume Duchenne. 5 8 9

Charles Bell provided rich descriptions of patients suffering from torticollis, including reports of the *geste antagoniste* , or “sensory trick” (although the first report probably occurred in Jäger's thesis, published in 1737, and the term itself was coined by Henry Meige and Louis Clement Feindel only in 1902). 8 11 However, the first photographic record was only taken in 1894 by Edóuard Brissaud, one of Jean-Martin Charcot's disciples. 5 8 9 12 13

Despite the fact that it is unclear if Gustave Doré was influenced by the medical advancements of his time, and if he intended to depict some form of cervical dystonia, his fine drawings certainly immortalized Rabelais' *torty colly* .

In conclusion, the art expressed in the literature of Rabelais, the poetry of Scarron, the drawings of Doré, and the photography of Brissaud immortalized the term torticollis: “Life imitates art far more than art imitates life” (Oscar Wilde).

## References

[1] Albanese A Bhatia K Bressman S B Phenomenology and classification of dystonia: a consensus update Mov Disord 2013 28 07 863 873 23649720 10.1002/mds.25475 PMC3729880

[2] Oppenheim H Über eine eigenartige Krampfkrankheit des kindlichen und jugendlichen Alters (Dysbasia lordotica progressiva, Dystonia musculorum deformans) Neurol Centrabl 1911 30 1090 1107

[3] Pearce J M Dystonia Eur Neurol 2005 53 03 151 152 15900098 10.1159/000085834

[4] Camargo C HF Teive H AG Evolution of the concept of dystonia Arq Neuropsiquiatr 2014 72 07 559 561. Doi: 10.1590/0004-282. Doi: × 20140056 25054990 10.1590/0004-282x20140056

[5] Newby R E Thorpe D E Kempster P A Alty J E A history of dystonia: Ancient to modern Mov Disord Clin Pract (Hoboken) 2017 4 04 478 485 10.1002/mdc3.12493 28920067 PMC5573933

[6] Maclean I Dr Rabelais's 500 year old prescription BMJ 1994 308 6932 803 804 10.1136/bmj.308.6932.803 8167483 PMC2540011

[7] Heath M J Rabelais. 1 Ed Tempe, Arizona Medieval & Renaissance texts & studies 1996

[8] Broussole E Laurencin C Bernard E Thobois S Danaila T Krack P Early illustrations of Geste Antagoniste in cervical and generalized dystonia. Tremor Other Hyperkinet Mov 2015. Doi: 10.7916/D8KD1. Doi: × 74 10.7916/D8KD1X74 PMC4582593 26417535

[9] Munts A G Koehler P J How psychogenic is dystonia? Views from past to present Brain 2010 133(Pt 5)1552 1564 10.1093/brain/awq050 20350935

[10] Rabelais F Doré G The Works of Rabelais, faithfully translated from the French with variorum notes and numerous illustrations by Gustave Doré Derby, England: The Moray Press 1854

[11] Bell C Case LXXIX. Spasmodic action on the sterno-cleido-mastoideus and trapezius muscles 1833. Duff Green, for the Register and Library of Medical and Chirurgical Science: Washington: p. 215

[12] Brissaud E Vingt-quatrième leçon. Tics et spasmes cloniques de la face Meige, H ed. *Leçons sur les maladies nerveuses: La Salpêtrière, 1893–1894* 1895. Masson: Paris: pp. 502–520.

[13] Marques P T Germiniani F MB Camargo C HF Munhoz R P Teive H AG Édouard Brissaud: distinguished neurologist and Charcot's pupil Arq Neuropsiquiatr 2018 76 07 490 493. Doi: 10.1590/0004-282. Doi: × 20180063 30066801 10.1590/0004-282X20180063

